# Evaluation of a Push-Pull System for the Management of *Frankliniella* Species (Thysanoptera: Thripidae) in Tomato

**DOI:** 10.3390/insects9040187

**Published:** 2018-12-07

**Authors:** Kara Tyler-Julian, Joe Funderburk, Mrittunjai Srivastava, Steve Olson, Scott Adkins

**Affiliations:** 1 Department of Entomology and Nematology, University of Florida, North Florida Research and Education Center, 155 Research Road, Quincy, FL 32351, USA; kara.tyler@ncf.edu (K.T.-J.); mrittunjai.srivistava@freshfromforida.com (M.S.); 2 Horticulture Department, University of Florida, North Florida Research and Education Center, 155 Research Road, Quincy, FL 32351, USA; smolson@ufl.edu; 3 US Horticultural Research Laboratory, USDA-ARS, Fort Pierce, FL 34945, USA; Scott.Adkins@ars.usda.gov

**Keywords:** companion plant, *Frankliniella occidentalis*, kaolin, thrips, UV-reflective mulch

## Abstract

A push-pull strategy for reducing populations of the thrips *Frankliniella occidentalis* (Pergande), *F. bispinosa* (Morgan) and *F. tritici* (Fitch) in tomato was evaluated. Push components consisted of ultraviolet (UV)-reflective mulch and foliar applications of kaolin and the pull component consisted of the companion plant *Bidens alba* (L.). Replicated field experiments were conducted in 2011 and 2012. Adult and larval thrips were reduced by UV-reflective mulch during early and mid-flowering of tomato. Spray applications of kaolin were effective in reducing adult and larval thrips during early, mid- and late-flowering. The pull effects of the *B. alba* companion plants were additive and sometimes interactive with the push effects of UV-reflective mulch and kaolin in reducing the adult males of each thrips species and the females of *F. bispinosa*. The strategy was not effective in reducing the adult females of *F. tritici* and *F. occidentalis*. In addition to attracting the *Frankliniella* species adults, the companion plants were hosts for the thrips predator *Orius insidiosus* (Say). The companion plants combined with UV-reflective mulch and kaolin proved effective as a push-pull system for suppressing flower thrips, including *F*. *occidentalis* which is a serious pest of tomato worldwide.

## 1. Introduction

Native to the southwestern United States, *Frankliniella occidentalis* (Pergande) (Thysanoptera: Thripidae) is now a common worldwide pest of tomato and other crops. It causes cosmetic damage from the feeding and oviposition injuries on the small fruits of tomato [1,2]. It is also the key vector of *Tomato spotted wilt virus* and other orthotospoviruses [3]. In Florida, the native congeners, *F. bispinosa* (Morgan) and *F. tritici* (Fitch), also inhabit the flowers of tomato and other fruiting vegetables [4] and these native species outcompete *F. occidentalis* [5].

The key natural enemy of flower thrips is the zoophytophagous insidious flower bug, *Orius insidiosus* (Say) (Hemiptera: Anthocoridae) [6]. This predator preys on thrips larvae and the adults of *F. occidentalis* over the adults of *F. bispinosa* and *F. tritici* [7,8]. Approximately one insidious flower bug for every 180 thrips is sufficient for suppression with thrips populations under control at a ratio of one per 40 thrips [6,9]. Usually, natural populations are not sufficient in tomato to provide control of thrips [10]. Allelochemicals in the plant’s trichomes deter the predator and alter its functional response in capturing thrips prey [11].

Calendar applications of broad-spectrum insecticides, such as pyrethroids, have been used for controlling thrips in fields. These applications were initially successful with their effects dwindling and reversing with increased use [12]. These chemicals eliminate the natural predators and the native species of thrips that would otherwise prevent *F. occidentalis* from attaining damaging levels in the field [12,13]. Applications of insecticides to control adult thrips in flowers do not prevent the transmission of orthotospoviruses [14,15]. Furthermore, the life history and genetic adaptations of *F. occidentalis* lead to the development of resistant populations [16]. Resistant populations of *F. occidentalis* combined with a lack of natural predators and competition from native thrips create a situation in which damaging populations are present in a field and cannot be controlled.

Ultraviolet (UV)-reflective mulch disrupts the ability of certain insects, including *F. occidentalis*, to find the host plant [17]. UV-reflective mulch reduces thrips numbers and the incidence of tomato spotted wilt disease, while increasing the yield of tomato [14]. Kaolin is an aluminosilicate mineral that is used on plants for pest control and protection from sun damage [18]. The modes of action include repelling light, impeding the ability of the insect to grasp the plant surface, deterring feeding and oviposition, impeding development and direct mortality [19,20,21,22,23]. Kaolin has been shown to reduce thrips on blueberries, onions and tomatoes [22,24,25].

There is potential to attract *O. insidiosus* into tomato fields using habitat management strategies such as companion plants [9]. A companion plant that is a host for *O. insidiosus* and a trap for flower thrips would be useful in a push-pull strategy [26]. *Helianthus annuus* L. previously was evaluated as a companion plant species in a push-pull system to manage flower thrips in pepper with kaolin and UV-reflective mulch evaluated as push components [9]. The objective of this research was to evaluate a push-pull system for managing flower thrips on tomatoes. Push components were UV-reflective mulch and foliar applications of kaolin and the pull component was the companion plant *Bidens alba* (L.) (Asteraceae).

## 2. Materials and Methods

### 2.1. Plot Establishment and Maintenance

Experiments on ‘Florida 47’ tomato and companion plants of *B. alba* were conducted in 2011 and 2012 at the North Florida Research and Education Center, University of Florida in Quincy (30° 32′ 52″ N, 84° 35′ 36″ W). Both tomato and companion plants were produced on raised plastic mulch beds 10 cm in height and 91.4 cm in width with a 1.83 m spacing between beds. Soil under the beds with tomato was fertilized with 204, 29 and 170 kg/ha of N, P and K, respectively. All beds were treated before mulch application with *S*-metolachlor (Syngenta Crop Protection LLC, Greensboro, NC, USA) at 1.2 kg active ingredient/ha for weed control. Each bed was irrigated with single trickle-tube with emitters spaced every 30 cm at a rate of 20,000 liters/ha/d. Weeds between the beds were additionally controlled by hand weeding and application of paraquat dichloride (Syngenta Crop Protection LLC) at 0.5 kg active ingredient/ha.

A split-split plot randomized complete block design with three replications was used each year to evaluate the separate and interactive effects of companion plants, mulch and kaolin. Whole-plot treatments were UV-reflective mulch and black mulch (Berry Plastics Corporation, Evansville, IN, USA), subplot treatments were kaolin (Surround WP, Engelhard Corp., Iselin, NJ, USA) and a control of no kaolin and sub-subplot treatments were *B. alba* companion plants and a control of no companion plants. The split-split plot treatment arrangement was used to reduce the inter-plot effects of UV-reflective mulch and kaolin on thrips movement [9,27]. Surround WP is 95% unprocessed mined mineral kaolin that is certified organic by the Organic Materials Review Institute) [28]. Sub-subplot size was 6 beds by 9m with the 4 inner beds of each sub-subplot consisting of one linear row of tomato with a 45-cm spacing between plants for a total of 80 plants per sub-subplot. Two rows of *B. alba* were transplanted into each of the two external beds with a 30-cm spacing within and between rows for a total of 128 companion plants per sub-subplot. No *B. alba* were planted within or between the four inner beds planted with tomato. Six-week old tomato and *B. alba* transplants were placed in the plastic mulch beds on 29 and 27 Mar in 2011 and 2012, respectively. Kaolin was applied to tomato plants twice weekly from 22 Apr to 3 Jun in 2011 and 30 Apr to 6 Jun in 2012 at a rate of 7.0 kg/ha using a CO_2_-backpack sprayer equipped with 5 hollow cone nozzles applying 405 liters/ha. 

### 2.2. Insect Sampling

Sampling for insects began on 26 Apr 2011 and 1 May 2012 within a few days of first flowering. Two samples of 10 flowers were randomly collected from the two outer tomato beds of each sub-subplot twice per week for 13 and 12 total sample dates in 2011 and 2012, respectively [29]. Two random samples of 10 *B. alba* flowers were collected on each sample date from each sub-subplot with companion plants. All flowers were placed in vials containing 70% ethanol. Thrips, *O. insidiosus* and other insects were extracted from flowers in each sample and identified to species, life stage and gender under a stereoscope with 40 to 100 × magnification. *Tomato spotted wilt virus* incidence was recorded in the two inner tomato beds of each sub-subplot as reported previously [27].

### 2.3. Statistical Analyses

The number of thrips larvae per adult *Frankliniella* species was determined on each 2011 and 2012 sample date for tomato and *B. alba* of each treatment. Ratios of < and > 1 indicated a declining and increasing population, respectively [30]. The ratio of total thrips (adults and larvae) per *O. insidiosus* was determined on each 2011 and 2012 sample date for tomato and *B. alba* of each treatment. Treatment effects each year in numbers of adult male and female *F. tritici*, adult male and female *F. occidentalis*, adult male and female *F. bispinosa*, larval thrips and adult and nymphal *O. insidiosus* per ten tomato flowers were analyzed each year using an analysis of variance for a randomized complete block design for a split-split plot treatment arrangement for data across sample date (PROC MIXED) [31]. The main and interactive effects in the model each year are shown in Table 1. The random effects included in the model were rep X mulch (date), rep X mulch X kaolin (date) and rep X mulch X kaolin X companion plant (date). Additional analyses were conducted to evaluate the effects of mulch, kaolin and companion plants on individual sample dates (PROC GLIMMIX) [31]. The random effects included in the model were rep, rep X mulch, rep X mulch X kaolin and rep X mulch X kaolin X companion plant. Treatment effects of mulch and kaolin each year in numbers of adult male and female *F. tritici*, adult male and female *F. occidentalis*, adult male and female *F. bispinosa*, larval thrips and adult and nymphal *O. insidiosus* per ten flowers in the *Bidens* companion plants were analyzed each year using an analysis of variance for a randomized complete block design for a split-plot treatment arrangement for data across sample date (PROC MIXED) [31]. The random effects included in the model were rep X mulch (date) and rep X mulch X kaolin (date). Data for all analyses was transformed to log10 (x + 1) and the main effects and their interactions were considered significant at α = 0.05.

## 3. Results

### 3.1. Abundance of Thrips and O. insidiosus in B. alba

The seasonal mean (SEM) per 10 *B. alba* flowers of *F. tritici*, *F. bispinosa* and *F. occidentalis* across all dates in 2011 was 100.7 (5.4), 0.43 (0.05) and 3.9 (0.27), respectively (n = 325 samples). The seasonal mean (SEM) per 10 *B. alba* flowers of *F. tritici*, *F. bispinosa* and *F. occidentalis* across all dates in 2012 was 33.4 (0.76), 83.4 (4.2) and 1.8 (0.17), respectively (n = 288 samples). The numbers of thrips larvae in *B. alba* flowers each year was less than 1% of the number of thrips adults. The seasonal mean (SEM) of thrips larvae per 10 *B. alba* flowers was 0.52 (0.07) and 1.8 (0.10) in 2011 and 2012, respectively (n = 325 and 288, respectively). The seasonal mean (SEM) per 10 *B. alba* flowers of adult and nymphal *O. insidiosus* was 2.7 (0.15) and 4.8 (0.19) across all dates in 2011 and 2012, respectively (n = 325 and 288, respectively). The ratio of the number of total thrips per *O. insidiosus* was 39 and 120 across date in 2011 and 2012, respectively. Therefore, the numbers of the predator relative to the numbers of thrips were sufficient to result in prey suppression. Adult and larval *Frankliniella* thrips and adult and nymphal *O. insidiosus* were > 98% of the total insects in the *B. alba* samples.

### 3.2. Effects of Mulch and Kaolin on Thrips and O. insidiosus in B. alba

There were no indications either year that mulch or kaolin treatments in the tomato crop affected the numbers of *F. occidentalis* or *O. insidiosus* in the flowers of the *B. alba* companion crop (data not shown). The main effect of mulch was not significant for female *F. occidentalis*, male *F. occidentalis* and total (adults and nymphs) *O. insidiosus* in 2011 (*F* = 0.4, 0.1 and 0.5, respectively; d. f. = 1, 28; *P* > 0.05) or in 2012 (*F* = 0.1, 1.0 and 0.1, respectively; d. f. = 1, 24; *P* > 0.05). The main effect of kaolin was not significant for female *F. occidentalis*, male *F. occidentalis* and total *O. insidiosus* in 2011 (*F* = 0.7, 0.0 and 0.0, respectively; d. f. = 1, 55; *P* > 0.05) and in 2012 (*F* = 0.1, 0.0 and 2.2, respectively; d. f. = 1, 48; *P* > 0.05).

The males of *F. tritici* in 2011 and the males of *F. bispinosa* in 2012 in the *B. alba* companion plant were reduced by application of kaolin in the tomato crop (*F* = 8.4 and 4.6, respectively; d. f. = 1, 55 and 1, 48, respectively; *P* = 0.005 and 0.04, respectively). The seasonal mean (SEM) per 10 *B. alba* flowers of the *F. tritici* males across all dates in 2011 was 38.4 (3.6) and 46.4 (4.6) in the kaolin and no kaolin plots, respectively (n = 325 samples), while the seasonal mean (SEM) per 10 *B. alba* flowers of the *F. bispinosa* males across all dates in 2012 was 30.1 (2.4) and 32.1 (2.5) in the kaolin and no kaolin plots, respectively (n = 288 samples). The males of *F. tritici* in 2012 and the males of *F. bispinosa* in 2011 in the *B. alba* companion plants were not affected by application of kaolin in the tomato crop (*F* = 0.3 and 2.0; d. f. = 1, 48 and 1,55; *P* > 0.05). The females of *F. tritici* in the *B. alba* companion plants were not affected by application of kaolin in the tomato crop in 2011 or 2012 (*F* = 2.2 and 0.2, respectively; d. f. = 1, 55 and 1, 48, respectively; *P* > 0.05). The females of *F. bispinosa* in the *B. alba* companion plants were not affected by application of kaolin in the tomato crop in 2011 or 2012 (*F* = 0.0 and 1.2, respectively; d. f. = 1, 55 and 1, 48, respectively; *P* > 0.05).

Neither the male nor female *F. tritici* in the *B. alba* companion plant were affected by the mulch treatments either year (data not shown). The main effect of mulch was not significant for female and male *F. tritici* in 2011 (*F* = 1.6 and 1.1, respectively; d. f. = 1, 28; *P* > 0.05) or in 2012 (*F* = 0.2 and 1.4, respectively; d. f. = 1, 24; *P* > 0.05). The males and females of *F. bispinosa* in the *B. alba* companion plants were not affected by the mulch treatments in 2011 (*F* = 1.2 and 0.1, respectively; d. f. = 1, 28; *P* > 0.05). The males and females of *F. bispinosa* in the *B. alba* companion plants were reduced by the mulch treatments in 2012 (*F* = 4.9 and 10.3, respectively; d. f. = 1, 24; *P* = 0.04 and 0.004, respectively). The seasonal mean (SEM) per 10 *B. alba* flowers of the *F. bispinosa* males in 2012 was 28.7 (2.1) and 33.6 (2.7) in the UV-reflective and black plots, respectively (n = 288 samples), while the seasonal mean (SEM) per 10 *B. alba* flowers of the *F. bispinosa* males in 2012 was 49.8 (3.5) and 54.8 (3.9) in the kaolin and no kaolin plots, respectively (n = 288 samples).

### 3.3. Effects of Mulch and Kaolin on Thrips and O. insidiosus in Tomato

Male and female *F. tritici* in 2011 and 2012, male and female *F. occidentalis* in 2011, male *F. bispinosa* in 2011 and 2012, female *F. bispinosa* in 2012 and thrips larvae in 2011 were significantly reduced by the UV-mulch (Table 1). Application of kaolin significantly reduced male and female *F. tritici* in 2011 and 2012, male and female *F. bispinosa* in 2011 and 2012, male *F. occidentalis* in 2011, female *F. occidentalis* in 2012 and thrips larvae in 2011. The mulch X kaolin interaction was significant for female *F. occidentalis* in 2011 and 2012, male *F. occidentalis* in 2011, female *F. tritici* in 2011, male *F. tritici* in 2011 and 2012 and thrips larvae in 2011. These interactions reflected the greater difference in magnitude in the reduction of the thrips where kaolin was applied to tomato on black mulch compared to where it was applied to tomato on UV-reflective mulch. There were no significant main or interactive effects of mulch and kaolin on the numbers of *O. insidiosus* in the tomato flowers.

**Figure 1:**
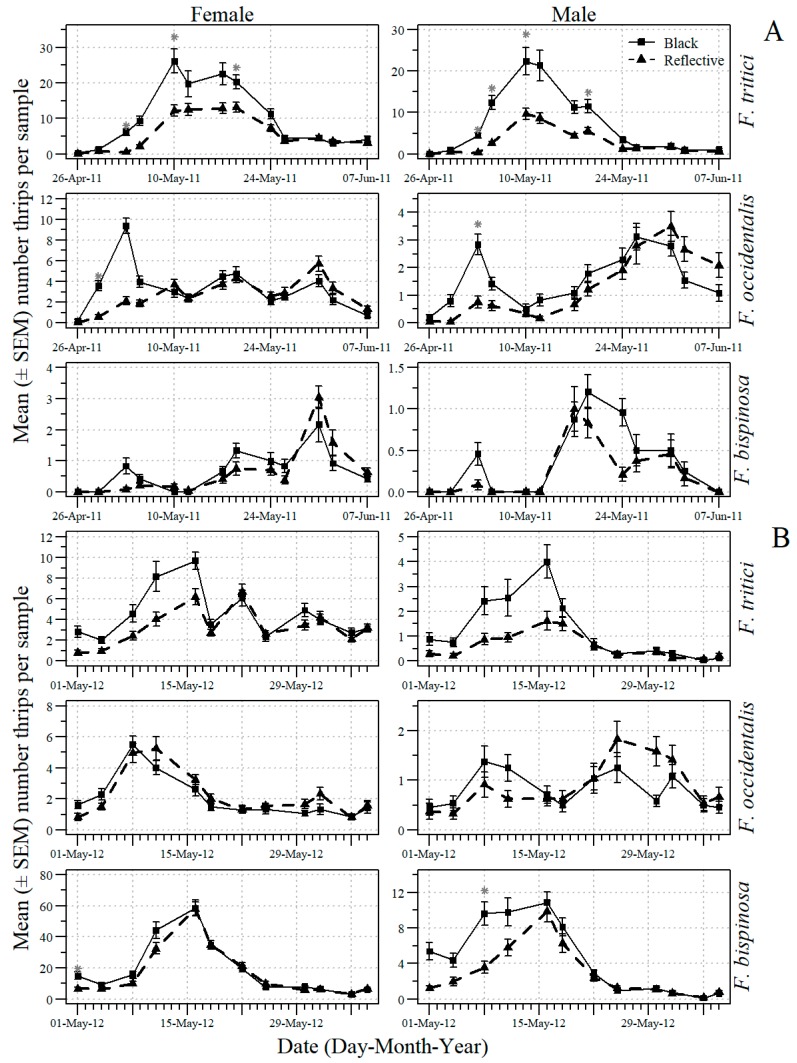
The mean number (+SEM) per 10 tomato flowers (n = 18 samples) of adult male and female *F. occidentalis*, *F. bispinosa* and *F. tritici* on each 2011 (**A**) and 2012 (**B**) sample date in the whole plot treatments of black and UV-reflective mulch for data pooled across kaolin and companion plant treatments in the experiments conducted in Gadsden County, Florida (* indicates significance beyond 95% level according to analysis of variance conducted for individual sample dates; d. f. = 1, 2).

**Figure 2:**
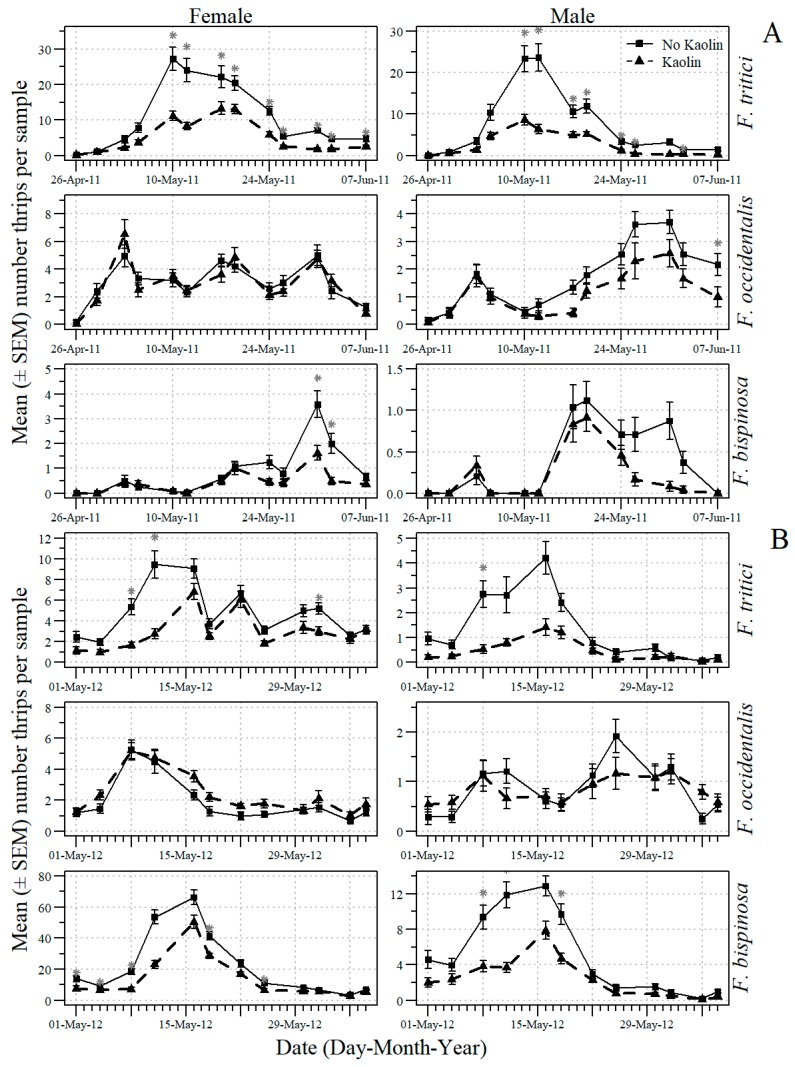
The mean number (+SEM) per 10 tomato flowers (n = 12 samples) of adult male and female *F. occidentalis*, *F. bispinosa* and *F. tritici* on each 2011 (**A**) and 2012 (**B**) sample date in the sub-plot treatments of no kaolin and kaolin for data pooled across companion plant treatments in the experiments conducted in Gadsden County, Florida (* indicates significance beyond 95% level according to analysis of variance conducted for individual sample dates; d. f. = 1, 4).

The date X mulch and the date X kaolin interactions were significant for the numbers of adult thrips in the tomato flowers (Table 1). For this reason, additional analyses were conducted to examine the effects of mulch type and kaolin application on individual dates. The mean number (SEM) for whole-plot mulch treatments of black and UV-reflective mulch on each sample date of male and female *F. tritici*, *F. bispinosa* and *F. occidentalis* in 2011 and 2012 are shown in Figure 1. The effect of UV-reflective mulch in reducing the numbers of adult thrips was significant on some 2011 and 2012 early and mid-flowering sample dates but not on late-flowering sample dates. The mean number (SEM) for subplot kaolin and no kaolin treatments on each sample date of male and female *F. tritici*, *F. bispinosa* and *F. occidentalis* in 2011 and 2012 are shown in Figure 2. The main effect of kaolin application in reducing the numbers of adult thrips was significant on some sample dates throughout 2011 and 2012.

### 3.4. Effects of Companion Plants on Thrips and O. insidiosus in Tomato

**Figure 3:**
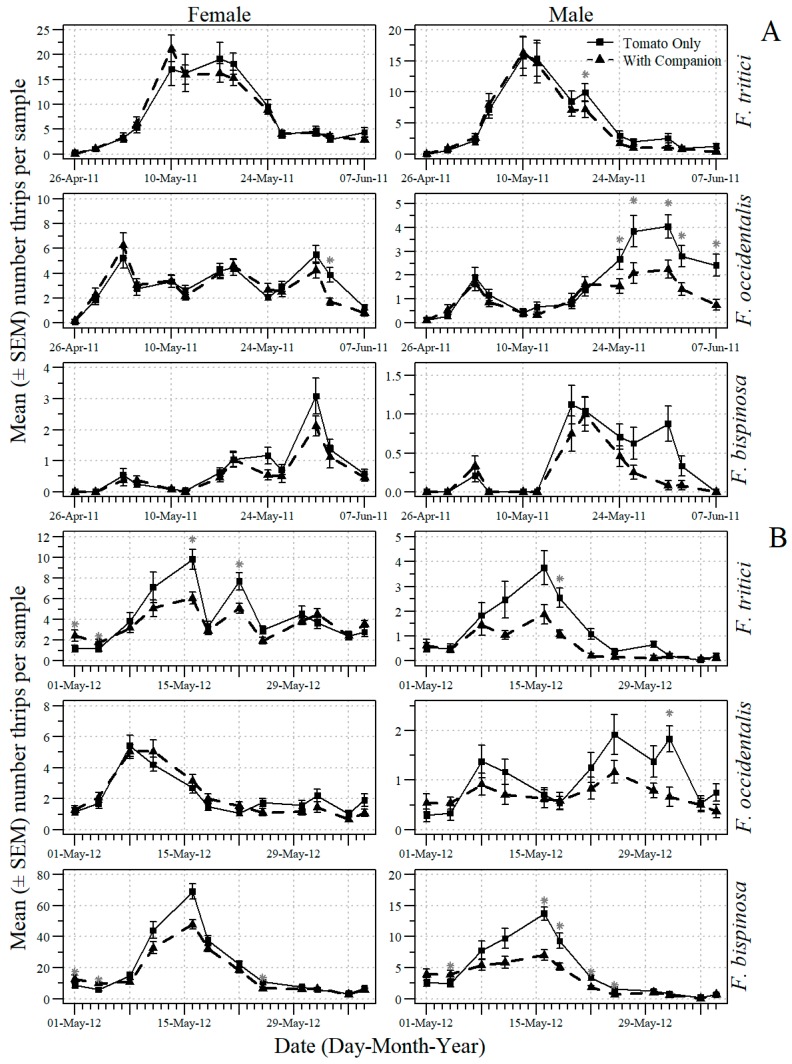
The mean number (+SEM) per 10 tomato flowers (n = 6 samples) of adult male and female *F. occidentalis, F. bispinosa and F. tritici* on each 2011 (**A**) and 2012 (**B**) sample date in the sub-subplot treatments of no companion plants and companion plants in the experiments conducted in Gadsden County, Florida (* indicates significance beyond 95% level according to analysis of variance conducted for individual sample dates; d. f. = 1, 8).

The companion plants were an effective tactic in reducing adult thrips in the tomato flowers (Table 1). There were significant main effects in 2011 and 2012 of the companion plants for data across sample date on populations of the males of each thrips species and for the females of *F. bispinosa*. Because the date X companion plant interaction usually was significant (Table 1), additional analyses were conducted to examine the effect of companion plant on individual dates. The mean number (SEM) of sub-subplot companion plant/no companion plant treatments on each sample date in 2011 and 2012 for male and female *F. tritici*, *F. bispinosa* and *F. occidentalis* are shown in Figure 3. The companion plants reduced the numbers of adult thrips of each species on some individual mid- and late-flowering sample dates in 2011 and 2012. 

The effect of the companion plants usually was additive and/or interactive with the effects of the UV-reflective mulch and kaolin in reducing adult thrips in the tomato flowers (Table 1). The main effects of companion plants and mulch were significant for *F. tritici* males in 2011 and 2012, *F. bispinosa* males in 2011 and 2012, *F. occidentalis* males in 2011 and *F. bispinosa* females in 2012. The mulch X companion plant interaction was significant for *F. tritici* and *F. bispinosa* males in 2012. These interactions reflected the greater difference in magnitude in the reduction of thrips due to the companion plants in the UV-mulch the black mulch plots. The main effects of companion plants and kaolin were significant for *F. occidentalis* males in 2011, *F. tritici* males in 2011 and 2012, *F. bispinosa* males in 2011 and 2012 and *F. bispinosa* females in 2011. The companion plants sometimes acted interactively with kaolin in reducing thrips numbers: the interactive effect of kaolin X companion plant was significant for male *F. tritici* in 2011 and 2012, male *F. bispinosa* in 2011 and female *F. tritici* in 2012. The significant mulch X kaolin X companion plant interaction for *F. tritici* males and females in 2011 indicated that the combined effects each tactic were not simply additive.

The main effect of companion plant on thrips larvae for data pooled across sample dates was significant in 2012 (Table 1). The mulch X companion plant interaction in 2012 reflected a greater number of thrips larvae in UV-reflective mulch treatments with companion plants compared with black mulch treatments with companion plants.

The effects of companion plants on *O. insidiosus* were not significant on individual sample dates in 2011 or 2012 (data not shown). The main effect of companion plant on total (adults + nymphs) *O. insidiosus* for data pooled across sample date was not significant in 2011 or 2012 (Table 1). The mulch X companion plant, kaolin X companion plant and mulch × kaolin × companion plant interactions were not significant for *O. insidiosus* in 2011 or 2012. The ratio of the numbers of total thrips per total *O. insidiosus* was 192 and 240 in 2011 and 2012, respectively. The numbers of the predator relative to the number of thrips were insufficient to result in prey suppression.

## 4. Discussion

The adults of *F. tritici*, *F. bispinosa* and *F. occidentalis* were abundant in the yellow flowers of tomato and the white flowers of *B. alba*. Terry [17] after reviewing the scientific literature concluded that adult *Frankliniella* thrips are attracted to low UV white, yellow and blue flowers for initial host finding and that the dominant wavelength remitted by the surface and the percentage reflectance at peak wavelengths is critical for finding hosts. Funderburk et al. [32] showed that *F. tritici* and *F. bispinosa* were most attracted to flowers of white *Lagerstroemia* species (Lithraceae) clones over the clones with flowers of other colors. 

The ratio of larvae to thrips adults was less than one in the tomato flowers for the season. Baez et al. [10] and Momol et al. [14] previously reported that *Frankliniella* thrips adults were abundant in the flowers of spring tomatoes in northern Florida but that populations declined. Tomato is a poor host because thrips must deal with defensive chemicals that reduce fitness [10]. The number of thrips larvae in the flowers of *B. alba* in this study was very low and their low numbers relative to high numbers of adults were evidence that *Frankliniella* thrips were not utilizing *B. alba* for breeding or that egg and larval survival was low. The flowers of *B. alba* undoubtedly served other functions, including being an important resource for food and mating. Mound [33] noted that the highly mobile thrips adults are attracted to and reach high numbers in the flowers of non-host species.

Numbers of adult thrips in the tomato flowers were reduced in the treatments with the *B. alba* companion plants compared to treatments without companion plants. Numerous studies showed that various host plants of *Orius* when used as companion plants resulted in reduced thrips in the crop [9,34,35,36,37,38]. The effect of the companion plants in reducing thrips in the tomato flowers was more consistent in this study across years and species for the adult males than the adult females. This result suggested that the males utilized the companion plants over the tomatoes as sites for forming mating swarms. Matteson and Terry [39] showed that the males of *F. occidentalis* most prefer low-UV white substrates over substrates of other colors for mating swarms and that the females left these aggregation sites soon after mating to exploit other flower resources for feeding and oviposition. 

In theory, companion plant species that are good hosts for *Orius* species can provide the benefit of biological control due to predation in the companion plant, or it can result from the companion plant serving as a host for increased populations of *Orius* that provide biological control in the crop. The adults and nymphs of *O. insidiosus* were abundant in the flowers of *B. alba* and their overall numbers in the companion crop relative to the numbers of prey were sufficient to predict suppression of thrips populations each year. Funderburk et al. [6], Reitz et al. [15] and Tyler-Julian et al. [9] reported that *O. insidiosus* suppressed field populations of thrips at a ratio of one predator per 180 prey. Funderburk et al. [32] showed that *O. insidiosus* aggregated with their thrips prey in a density-dependent manner: the adults by preferring the *Lagerstroemia* species clones also preferred by the thrips and the nymphs by direct tracking or as a function of increased prey and fecundity. White-flowered clones of *Lagerstroemia* were best for preference and buildup of *O. insidiosus* populations. The thrips adults may have preferred the white flowers of *B. alba* over the yellow tomato flowers, thereby reducing the number of thrips adults in the tomato flowers. Overall, our results in this study suggest that the effect of the companion plants in reducing thrips in the tomatoes were due to the high attractiveness of the white flowers to the thrips adults where they suffered from predation by *Orius*.

The adults and nymphs of *O. insidiosus* were very low in the tomato flowers in this study and the *B. alba* companion plants did not result in an increase in the number of *Orius* in the tomato crop. Baez et al. [7] and Momol et al. [14] previously reported that tomato was a very poor host for *O. insidiosus*. Tomatoes hamper the predator in several ways: searching ability is reduced because of the presence of plant trichomes, adults and nymphs suffer high mortality after collecting viscous tomato material on their legs and the plant is unsuitable for nymphal development and female reproduction [11]. 

Numbers of adult and larval thrips were reduced by UV-reflective mulch during early and mid-flowering of tomato. Momol et al. [14] previously reported that UV-mulch was effective in reducing the numbers of *F. bispinosa*, *F. tritici* and *F. occidentalis* during early and mid-flowering. Thrips numbers were not affected during late flower of tomato once the plants grew and the leaves covered the UV-reflective mulch. Momol et al. [14] showed that the effects of UV-reflective mulch on the thrips vectors also reduced the incidence of plants infected with *Tomato spotted wilt virus*. Disease incidence was reduced by UV mulch, kaolin and companion plants in this study, as we previously reported in Tyler-Julian et al. [27]. Numbers of *O. insidiosus* in the tomato flowers were not affected by UV mulch, kaolin, or companion plants in this study, undoubtedly because the tomatoes were not a host as previously discussed. Reitz et al. [15] and Tyler-Julian et al. [9] showed that the UV-reflective mulch disrupted host finding by *O. insidiosus* in pepper, which is a good host for the predator. 

Spray applications of kaolin were effective in reducing adult and larval thrips in this study. The effects were evident during early, mid- and late-flowering. Reitz et al. [25] previously reported the effectiveness of kaolin in reducing *Frankliniella* thrips in tomato. The effects of mulch and kaolin were sometimes additive, as evidenced by significant simultaneous main effects. There were sometime interactive effects between kaolin and UV-reflective mulch in reducing thrips populations. Tyler-Julian et al. [9] reported that thrips numbers were reduced by kaolin application during early flowering of pepper but that numbers increased on later sample dates due to negative effects of kaolin on *O. insidiosus* numbers. Numbers of *O. insidiosus* were very low in tomato flowers and no such effects were observed in this study.

Push-pull strategies seek to maximize efficacy of behavior-manipulating stimuli through the additive and synergistic effects of integrating their use [26]. Simulation models predicted that companion plants with the characteristics of strong attraction and arrestment offer the best opportunities for trapping pests that use visual cues to find host plants [40]. In addition, plants that are good hosts for key natural enemies provide the additional benefit of biological control. In this study, the pull effects of companion plants were additive or interactive with the push effects of UV-reflective mulch and kaolin in reducing male thrips in tomato each year. This push-pull strategy was less effective in reducing female thrips in tomato. This suggests that the females left the flowers of *B. alba* soon after mating and they were less vulnerable than the males to predation from *Orius*.

Tyler-Julian et al. [9] previously evaluated sunflower (*Helianthus annuus* L.) as a companion plant with UV-reflective mulch and kaolin in a push-pull system to reduce *F. bispinosa* in pepper (*Capsicum annuum* L.). There was a rapid buildup of *F. bispinosa* populations in the flowers of both the companion plant and the crop, but they were soon suppressed to near extinction due to predation. Predator populations persisted in pepper and sunflower for the remainder of each season in numbers sufficient to prevent further buildup of thrips populations. The overall dynamic relationship of time to prey suppression and near extinction of populations was not much altered by the presence of the companion plant. In that system in which the crop and the companion plant species were excellent hosts for the thrips and the predator, the companion plant did not act additively or interactively with kaolin or UV-reflective mulch in reducing *F. bispinosa* and increasing *Orius* species in the crop.

Species of *Bidens* grow as an annual any time of the year throughout Florida and the species grow readily in disturbed habitats of agricultural landscapes. We found that *B. alba* is easily established as a companion plant prior to the critical flowering periods of vulnerability to *F. occidentalis* and a single planting is readily maintained beyond flowering of fruiting vegetables. Its ability to re-establish as a ‘weedy’ companion for future crops with minimal effort by the producer is very possible. The species begins flowering within weeks and new shoots provide for a continuous supply of flowers. Needham [41] conducted a study of the arthropod species that form a community of herbivores and natural enemies in the flowers of *B. pilosa*, including the thrips and *O. insidiosus* that he noted were ubiquitous and occurred throughout the year in Florida. He produced a systematic list of herbivore species, mostly Diptera, Lepidoptera and Hemiptera and of natural enemy species, mostly Hymenoptera. We found little information in the scientific literature on the population dynamics of arthropods in species of *Bidens*.

Because species of *Bidens* are inhabited by a community of herbivores and natural enemies, it is possible that there are important intraguild and other complex food web interactions between thrips, *O. insidiosus* and other arthropods. Our studies confirmed Needham’s [41] observations that thrips are overwhelmingly abundant in the flowers of *Bidens*. The studies of Funderburk et al. [6], Ramachandran et al. [42], Reitz et al. [15] and Hansen et al. [29] revealed predator-prey dynamics of *O. insidiosus* and flower thrips and they revealed the adaptive ability of *O. insidiosus* to exploit thrips prey and cause near extinction of *Frankliniella* species populations. The sheer numbers of thrips and *O. insidiosus* relative to the other insects in the flowers of *B. alba* in this study suggested that there were no food web or intraguild interactions that interfered with the predator-prey relationships between thrips and *O. insidiosus*.

## 5. Conclusions

Tomato was a very poor host for *F. occidentalis*, *F. tritici*, and *F. bispinosa* and populations declined due to poor reproduction. Even so, tomato was vulnerable to damage from *F. occidentalis* (and orthotospoviruses, as previously reported [27]). Predation from *O. insidiosus* was less important in tomato than in the *B*. alba companion plant. The companion plants of *B. alba* were a pull in this study, attracting the thrips and partially arresting the thrips males away from the tomato crop. The companion plants were a host for *O. insidiosus* and the thrips pulled into the companion plant were exposed to predation from this key natural enemy. We tested separately and combined UV-reflective mulch and applications of kaolin as push factors with and without companion plantings of *B. alba* as the pull factor. The UV-reflective mulch was effective in reducing thrips numbers (and the incidence of tomato spotted wilt disease as previously reported [27]) from early to midseason. The kaolin applications were effective in reducing populations any time after applications were made. The companion plants of *B. alba* combined with UV-reflective mulch and kaolin applications proved effective as a push-pull system in tomato for suppressing *F. occidentalis*, a serious worldwide pest and key vector of *Tomato spotted wilt virus*. This study serves as a proof of concept of the push-pull system under investigation and additional research is needed to determine its applicability in different agricultural production systems.

## Figures and Tables

**Table 1:** Mean number per 10 tomato flowers (SEM) of adult male and female *F. occidentalis*, adult male and female *F. tritici*, adult male and female *F. bispinosa*, *Frankliniella* species larvae and total *O. insidiosus* (adults and nymphs) in mulch, kaolin and companion plant treatments for sample data pooled across 13 dates in 2011 (*n* = 78) and 12 dates (*n* = 72) in 2012 in push-pull experiments conducted in Gadsden County, Florida.

Treatment	Mean no. per 10 Tomato Flowers (SEM)							
	*F. occidentalis*		*F. tritici*		*F. bispinosa*		Thrips Larvae	*O. insidiosus*
	Males	Females	Males	Females	Males	Females		
2011								
Black mulch	2.1 (0.2)	3.1 (0.3)	12.0 (1.6)	16.7 (2.0)	0.6 (0.1)	1.1 (0.2)	6.8 (0.9)	0.10 (0.05)
Black mulch and companion plants	1.5 (0.2)	3.1 (0.4)	10.1 (1.5)	12.2 (1.3)	0.7 (0.1)	0.3 (0.1)	5.7 (0.7)	0.19 (0.06)
Black mulch and kaolin	1.5 (0.2)	3.7 (0.4)	3.0 (0.5)	5.1 (0.7)	0.2 (0.1)	0.5 (0.1)	4.1 (0.6)	0.06 (0.03)
Black mulch and companion plants and kaolin	1.1 (0.2)	3.4 (0.4)	3.7 (0.6)	6.9 (0.9)	0.3 (0.1)	0.3 (0.1)	4.5 (0.7)	0.14 (0.05)
UV mulch	2.0 (0.3)	3.0 (0.3)	3.9 (0.5)	6.5 (0.8)	0.5 (0.1)	0.8 (0.2)	5.3 (0.9)	0.12 (0.04)
UV mulch and companion plants	1.2 (0.2)	3.0 (0.3)	3.7 (0.6)	8.3 (1.0)	0.2 (0.1)	0.7 (0.2)	4.9 (0.8)	0.19 (0.05)
UV mulch and kaolin	1.3 (0.3)	2.5 (0.3)	2.4 (0.5)	4.4 (0.6)	0.2 (0.1)	0.5 (0.1)	3.9 (0.6)	0.13 (0.05)
UV mulch and companion plants and kaolin	0.6 (0.1)	2.2 (0.2)	1.6 (0.3)	4.3 (0.5)	0.1 (0.0)	0.5 (0.1)	3.7 (0.5)	0.09 (0.03)
	Analysis of variance *F*-value							
Date (12, 26 df)	20.0 ***	22.8 ***	102.6 ***	70.3 ***	20.1 ***	24.6 ***	81.4 ***	3.4 **
Rep (2, 26 df)	4.6 *	5.0 *	1.3	0.5	0.1	1.3	0.7	1.6
Mulch (1, 26 df)	11.2 **	7.8 **	111.9 ***	48.1 ***	8.2 **	0.5	13.2 **	0.2
Date X mulch (12, 26 df)	3.7 **	8.1 ***	7.2 ***	4.6 ***	2.2 *	2.9 **	2.0	0.5
Kaolin (1, 52 df)	39.3 ***	1.1	278.1 ***	169.0 ***	10.1 **	24.0 **	24.9 ***	1.7
Date X kaolin (12, 52 df)	1.7	1.7	6.1 ***	4.6 ***	2.3*	3.9 ***	1.7	0.5
Mulch X kaolin (1, 52 df)	0.8	5.4*	35.0 ***	21.3 ***	0.1	3.7	8.0 **	0.0
Date X mulch X kaolin (12, 52 df)	0.5	1.7	1.6	1.9*	0.9	1.1	1.3	0.9
Companion plant (1, 104 df)	21.3 ***	2.0	5.8*	2.0	12.1 ***	7.3 **	0.6	2.2
Date X companion plant (12, 104 df)	3.0 **	1.8	2.4 **	0.9	2.5 **	0.9	0.3	0.4
Mulch X companion plant (1, 104 df)	0.3	0.5	0.0	0.2	0.1	0.7	0.2	0.8
Date X mulch X companion plant (12, 104 df)	0.5	1.4	1.7	0.3	0.9	1.0	2.0*	0.4
Kaolin X companion plant (1, 104 df)	0.5	1.4	7.2 **	2.8	12.4 ***	0.2	1.4	0.9
Date X kaolin X companion plant (1, 104 df)	0.5	0.9	1.6	1.1	2.6 **	1.2	0.5	1.0
Mulch X kaolin X companion plant (1, 104 df)	0.1	0.1	9.3 **	12.8 ***	3.6	0.3	0.6	0.3
Date X mulch X kaolin X companion plant (12, 104 df)	0.4	0.8	1.7	1.3	1.5	1.2	0.7	1.7
2012								
Black mulch	0.9 (0.1)	1.6 (0.2)	2.6 (0.4)	6.9 (0.7)	7.6 (0.9)	26.4 (3.3)	8.1 (0.9)	0.14 (0.05)
Black mulch and companion plants	0.6 (0.1)	1.6 (0.2)	1.1 (0.2)	4.4 (0.3)	4.7 (0.6)	20.9 (2.2)	6.1 (0.7)	0.18 (0.05)
Black mulch and kaolin	1.1 (0.2)	2.7 (0.3)	0.7 (0.1)	3.3 (0.4)	3.5 (0.5)	15.7 (2.2)	7.3 (0.9)	0.14 (0.05)
Black mulch and companion plants and kaolin	0.8 (0.1)	2.5 (0.3)	0.4 (0.1)	3.4 (0.3)	2.4 (0.3)	13.2 (1.6)	7.7 (0.8)	0.14 (0.04)
UV mulch	1.2 (0.2)	2.2 (0.3)	1.0 (0.2)	4.1 (0.4)	4.6 (0.7)	22.2 (2.9)	7.4 (1.1)	0.19 (0.06)
UV mulch and companion plants	0.8 (0.1)	2.4 (0.3)	0.6 (0.1)	3.8 (0.3)	3.2 (0.4)	18.2 (2.1)	10.3 (1.3)	0.19 (0.06)
UV mulch and kaolin	0.9 (0.2)	2.4 (0.2)	0.4 (0.1)	2.5 (0.3)	2.2 (0.4)	14.8 (2.1)	6.8 (0.8)	0.13 (0.04)
UV mulch and companion plants and kaolin	0.6 (0.1)	2.1 (0.2)	0.4 (0.1)	2.7 (0.3)	1.7 (0.2)	12.0 (1.5)	7.9 (0.9)	0.15 (0.06)
	Analysis of variance *F*-value							
Date (11, 24 df)	3.6 ***	26.3 ***	19.9 ***	25.4 ***	83.7 ***	95.0 ***	24.0 ***	6.2 ***
Rep (2, 24 df)	0.7	0.9	1.9	5.9 **	0.3	10.0 ***	4.8*	1.8
Mulch (1, 24 df)	0.2	2.2	21.8 ***	24.2 ***	37.5 ***	7.7 **	0.0	0.0
Date X mulch (11, 24 df)	1.2	2.1	2.1	3.0 **	5.5 ***	2.4 *	0.9	0.4
Kaolin (1, 48 df)	0.0	19.5 ***	74.6 ***	72.7 ***	91.6 ***	111.1 ***	0.2	1.0
Date X kaolin (11, 48 df)	1.9	0.9	4.7 ***	3.3 **	3.5*	3.6 **	1.9	0.7
Mulch X Kaolin (1, 48 df)	8.2 **	7.7 **	13.0 ***	2.7	0.3	0.8	0.6	0.1
Date X mulch X kaolin (11, 48 df)	1.0	0.9	2.3 *	1.8	0.8	0.7	0.2	1.1
Companion plant (1, 96 df)	10.3 **	0.7	26.2 ***	0.0	11.7 ***	4.1*	4.0*	0.3
Date X companion plant (11, 96 df)	2.0 *	1.7	2.7 **	2.9 **	4.3 ***	3.7 ***	1.9 *	0.8
Mulch X companion plant (1, 96 df)	0.3	0.0	6.6 **	3.4	4.1 *	0.0	6.6 **	0.1
Date X mulch X companion plant (11, 96 df)	0.8	0.9	1.5	0.9	1.0	1.1	1.2	0.5
Kaolin X companion plant (1, 96 df)	0.1	2.5	6.2 **	6.1 **	2.1	1.1	0.0	0.1
Date X kaolin X companion plant (11, 96 df)	1.0	0.5	1.7	0.7	1.2	0.9	0.9	0.5
Mulch X kaolin X companion plant (1, 96 df)	0.3	0.7	0.2	2.4	0.0	0.0	6.6 **	0.1
Date X mulch X kaolin X companion plant (11, 96 df)	0.7	0.7	0.9	0.8	0.7	0.7	1.3	0.5

Analysis of variance *F*-values are included of the main and interactive effects of mulch (whole plots), kaolin (subplots) and companion plants (sub-subplots). * *P* < 0.05; ** *P* < 0.01; *** *P* < 0.001.
